# Differentiation of human induced pluripotent stem cells into Leydig-like cells with molecular compounds

**DOI:** 10.1038/s41419-019-1461-0

**Published:** 2019-03-04

**Authors:** Xianwu Chen, Chao Li, Yong Chen, Haitao Xi, Shenzhi Zhao, Leikai Ma, Zhangye Xu, Zhao Han, Junzhao Zhao, Renshan Ge, Xiaoling Guo

**Affiliations:** 1 Center of Scientific Research, the Second Affiliated Hospital and Yuying Children’s Hospital of Wenzhou Medical University, Wenzhou, Zhejiang China; 2 Reproductive Medicine Center, the Second Affiliated Hospital and Yuying Children’s Hospital of Wenzhou Medical University, Wenzhou, Zhejiang China; 3 Department of Gynecology and Obstetrics, the Second Affiliated Hospital and Yuying Children’s Hospital of Wenzhou Medical University, Wenzhou, Zhejiang China; 4 Department of Neurology, the Second Affiliated Hospital and Yuying Children’s Hospital of Wenzhou Medical University, Wenzhou, Zhejiang China

## Abstract

Leydig cells (LCs) play crucial roles in producing testosterone, which is critical in the regulation of male reproduction and development. Low levels of testosterone will lead to male hypogonadism. LC transplantation is a promising alternative therapy for male hypogonadism. However, the source of LCs limits this strategy for clinical applications. Thus far, others have reported that LCs can be derived from stem cells by gene transfection, but the safe and effective induction method has not yet been reported. Here, we report that Leydig-like cells can be derived from human induced pluripotent stem cells (iPSCs) using a novel differentiation protocol based on molecular compounds. The iPSCs-derived Leydig-like cells (iPSC-LCs) acquired testosterone synthesis capabilities, had the similar gene expression profiles with LCs, and positively expressed Leydig cell lineage-specific protein markers LHCGR, STAR, SCARB1, SF-1, CYP11A1, HSD3B1, and HSD17B3 as well as negatively expressed iPSC-specific markers NANOG, OCT4, and SOX2. When iPSC-LCs labeled with lipophilic red dye (PKH26) were transplanted into rat testes that were selectively eliminated endogenous LCs using EDS (75 mg/kg), the transplanted iPSC-LCs could survive and function in the interstitium of testes, and accelerate the recovery of serum testosterone levels and testis weights. Collectively, these findings demonstrated that the iPSCs were able to be differentiated into Leydig-like cells by few defined molecular compounds, which may lay the safer groundwork for further clinical application of iPSC-LCs for hypogonadism.

## Introduction

Leydig cells (LCs), which reside in the testis interstitium, were first identified in 1850 by Franz Leydig, and the name Leydig cells was coined after him. Eutherian mammals develop at least two types of LCs: fetal Leydig cells and adult Leydig cells (ALCs) in the fetal and adult testis, respectively^1^. The ALC population ultimately develops from undifferentiated mesenchymal-like stem cells. In vivo, the developmental process consists of four steps: stem Leydig cells (undifferentiated mesenchymal-like stem cells), progenitor Leydig cells, immature Leydig cells (ILCs), and ALCs^2–5^.

Testosterone synthesized by LCs is essential for the physiological functions of the male reproductive system^6,7^. Male hypogonadism is a symptomatic clinical syndrome caused by testosterone deficiency, which is characterized by mood disturbance and fatigue, sexual dysfunction, decreased muscle mass and strength, decreased lean body mass and bone mineral density, and increased visceral fat^8–10^. These changes can be partially overcome by exogenous testosterone replacement therapy^11,12^. However, it disrupts the hypothalamic–pituitary–testicular axis, and may increase the risks of cardiovascular disorders and prostate tumorigenesis^13,14^. In addition, as physiological requirements of testosterone vary in individuals^15^, it is difficult for exogenous testosterone supplementation to meet the requirements of individualized treatment. Therefore, it becomes necessary to explore a new therapy for testosterone supplementation in a physiological pattern. LC transplantation is an ideal physiological and long-acting system for the testosterone delivery^16^. However, LCs account for only ~ 2–4% of the total testicular cell population in adult human testes^17^. Moreover, LCs are terminally differentiated cells with a limited capacity to proliferate^4^, thereby limiting the efficacy of LC transplantation therapy.

Stem cell-derived Leydig cell transplantation may be a promising alternative therapy for male hypogonadism. Although several studies have attempted to differentiate stem cells, such as mesenchymal stem cells^18,19^, embryonic stem cells (ESCs)^20–22^, and induced pluripotent stem cells (iPSCs)^23^ into steroid-producing cells by exogenous gene transfection, it is not so safe for further clinical application.

In this study, we present a small-molecule-based strategy for the efficient induction of LCs from iPSCs. We found that differentiation toward Leydig-like cells was induced by few defined molecular compounds. Transplantation of these Leydig-like cells into an animal model treated with ethylene dimethanesulfonate (EDS)^24^ could promote the recovery of serum testosterone levels and reproductive organ weights. Our findings will provide new insight into the development of cell replacement therapies for hypogonadism.

## Results

### Identification of iPSCs

iPSCs were often cultured by clonal growth on 1% Matrigel-coated dishes in E8 medium (Fig. S1a). Karyotype analysis revealed that iPSCs maintained a normal karyotype of 46XY (Fig. S1b). Immunofluorescence assay (Fig. 2a), reverse transcription-polymerase chain reaction (RT-PCR) assay (Fig. 3a), and western blotting (Fig. 5a) demonstrated that iPSCs could express pluripotent markers such as NANOG, OCT4, and SOX2 in vitro. To further confirm pluripotency in vitro, iPSCs were subcutaneously injected into severe combined immune deficiency (SCID) mice. Teratomas containing three germ layers (endoderm, ectoderm, and mesoderm) were observed by 6 weeks after injection (Fig. S1c).

### Differentiation of iPSCs into Leydig-like cells (iPSC-LCs)

**Fig. 1:**
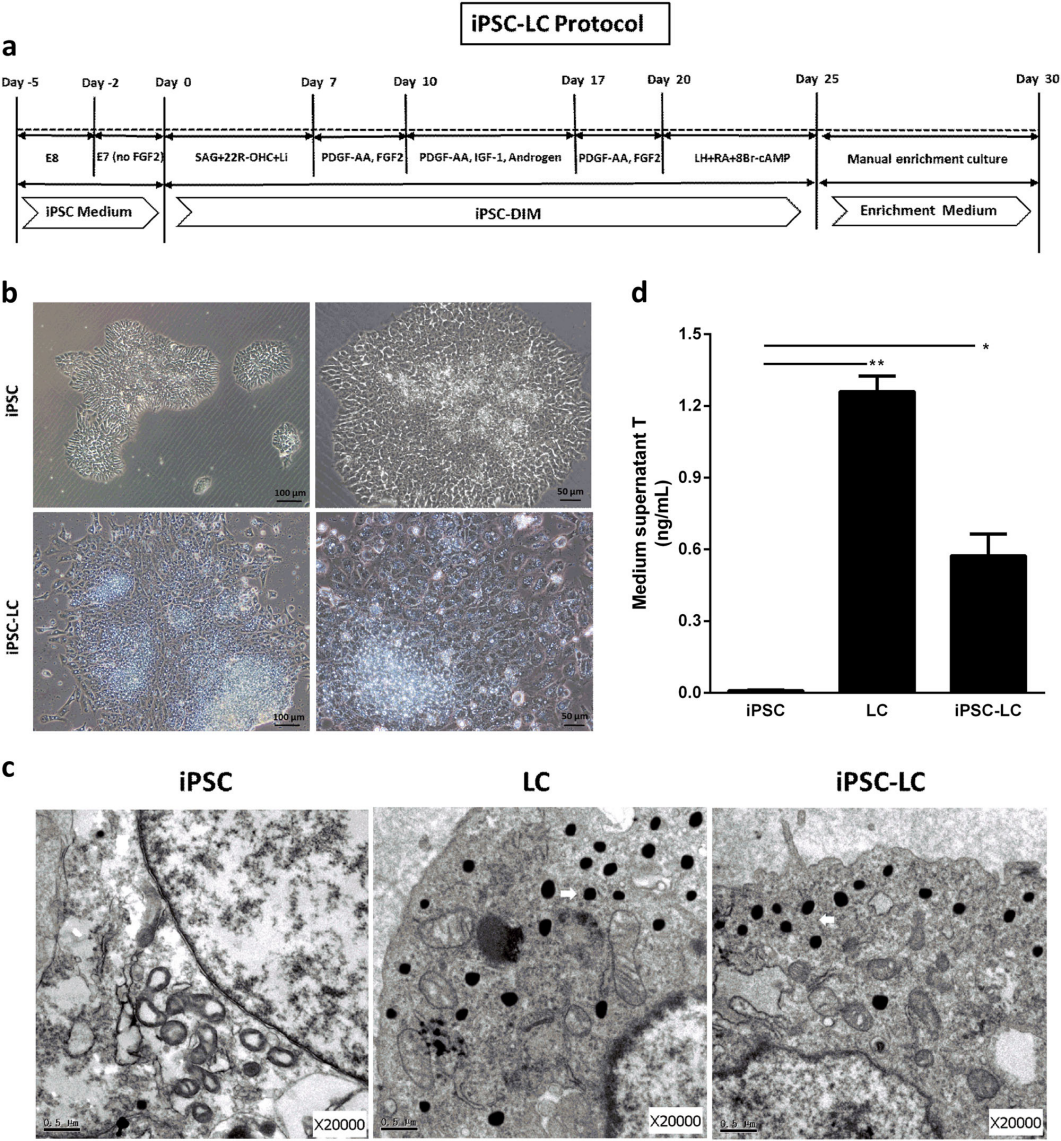
Differentiation-induced pluripotent stem cells (iPSCs) into Leydig-like cells (iPSC-LCs) based on molecular compounds. **a** The schematic illustration of differentiation protocol of iPSC-LCs. **b** Morphological changes in the differentiation of iPSC-LCs under inverted microscope on day 25. **c** The transmission electron microscopy (TEM) in different group cells. White arrows point to lipid droplets. **d** Medium testosterone (T) levels in different groups by radioimmunoassay. Mean ± SE, *n* = 5. ^*^P < 0.05, ^**^P < 0.01, designate significant differences

Because a prerequisite for iPSC differentiation is the shutdown of the self-renewal machinery, iPSCs were pretreated in E7 medium (without FGF2) for 2 days to encourage the spontaneous differentiation^25^. Then, we switched iPSCs in a differentiation-inducing medium (iPSC-DIM) with the sequential addition of defined molecular compounds at specific times for 25 days in order to favor the differentiation and proliferation. iPSC-LCs were manually enriched through scraping away clonal iPSC-like cells for the subsequent assays. The schematic illustration is displayed in Fig. 1a. The necessity and optimum dose of all defined molecular compounds, which were required to induce the differentiation of iPSCs into Leydig-like cells, were screened and analyzed by comparing the secreted testosterone levels of iPSC-LCs, which were induced using different molecular compound combinations (Table S3 and S4). iPSCs often showed adherent clonal growth, and the clones gradually became larger with time. After differentiation on day 25, the partial Leydig-like cells predominantly exhibited ellipse shapes and tended to group together to form clusters but the adherent cell clones gradually withered (Fig. 1b). In addition, the results of transmission electron microscopy showed that both iPSC-LC and LC had many lipid droplets present in the cytoplasm, which were necessary for testosterone production, but there was a little lipid droplets found in iPSC (Fig. 1c). Under the stimulus of 10 ng/mL luteinizing hormone (LH) for 3 h, the enrichment iPSC-LCs could secrete testosterone into the medium, which was more than iPSCs (*P* < 0.05, almost undetectable) but less than LCs (*P* < 0.01) (Fig. 1d). These results demonstrated that this protocol is able to efficiently differentiate partial iPSCs into Leydig-like cells.

### Identification of Leydig-like cells derived from iPSCs (iPSC-LCs)

**Fig. 2:**
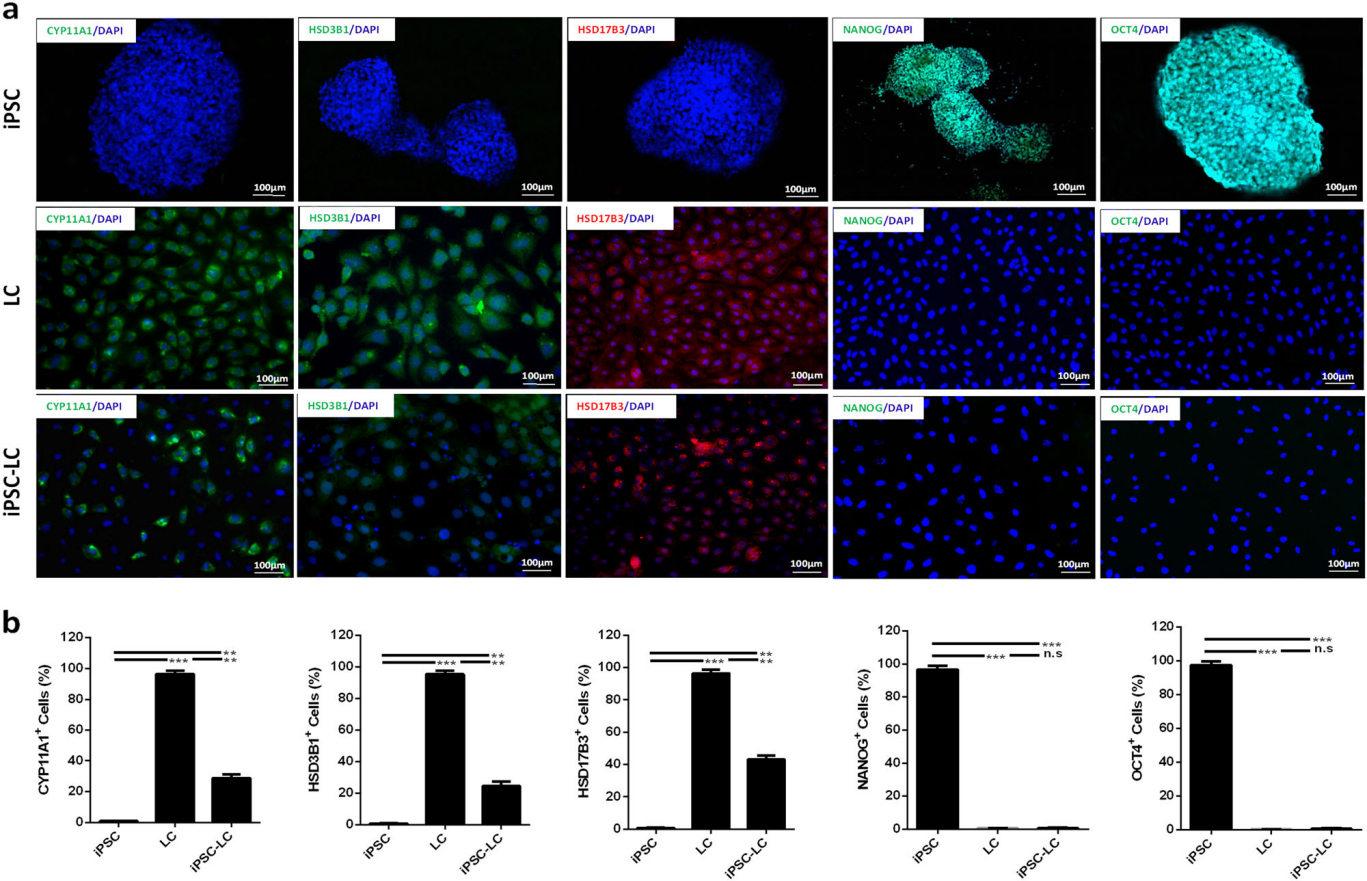
Identification of Leydig-like cells derived from induced pluripotent stem cells (iPSC-LCs) by immunofluorescence assays. **a** The detection of protein biomark expressions of Leydig cells or iPSCs using immunofluorescence assays in iPSCs, LCs, and iPSC-LCs. **b** The statistical analysis of immunofluorescence. Mean ± SE, *n* = 5. ^**^P < 0.01, ^***^P < 0.001 designate significant differences. n.s > 0.05 designates no significant difference

After 30 day differentiation, the immunofluorescence assay was used to characterize the expression of Leydig cell or iPS cell protein biomarkers in the enrichment iPSC-LCs. The results showed that iPSC-LCs could partially positively express Leydig cell biomarkers such as CYP11A1, HSD3B1, and HSD17B3, but negatively express iPS cell biomarkers NANOG and OCT4. Undifferentiated iPSCs negatively express CYP11A1, HSD3B1, and HSD17B3, but positively express NANOG and OCT4. LCs strongly express CYP11A1, HSD3B1, and HSD17B3, but negatively express NANOG and OCT4 (Fig. 2a). The statistical data on the positive percentages of biomarker expressions were shown in Fig. 2b. The results showed that the percentages of positive cells expressing Leydig cell markers such as CYP11A1, HSD3BI, and HSD17B3 in iPSCs were 0.95%, 0.91%, 0.96%, respectively, which were lower than these of LCs (98.53%, 97.81%, 98.70%) and iPSC-LCs (28.42%, 24.42%, 42.18%). In addition, the percentages of positive cells expressing iPS cell markers such as NANOG and OCT4 in iPSCs were 98.91% and 99.82%, which were higher than these of LCs (0.88% and 0.65%) and iPSC-LCs (0.98% and 1.02%). These results further illuminated that this method based on molecular compounds could differentiate partial iPSCs into Leydig-like cells.

### Identification of Leydig-like cells derived from iPSCs  (iPSC-LCs)

**Fig. 3:**
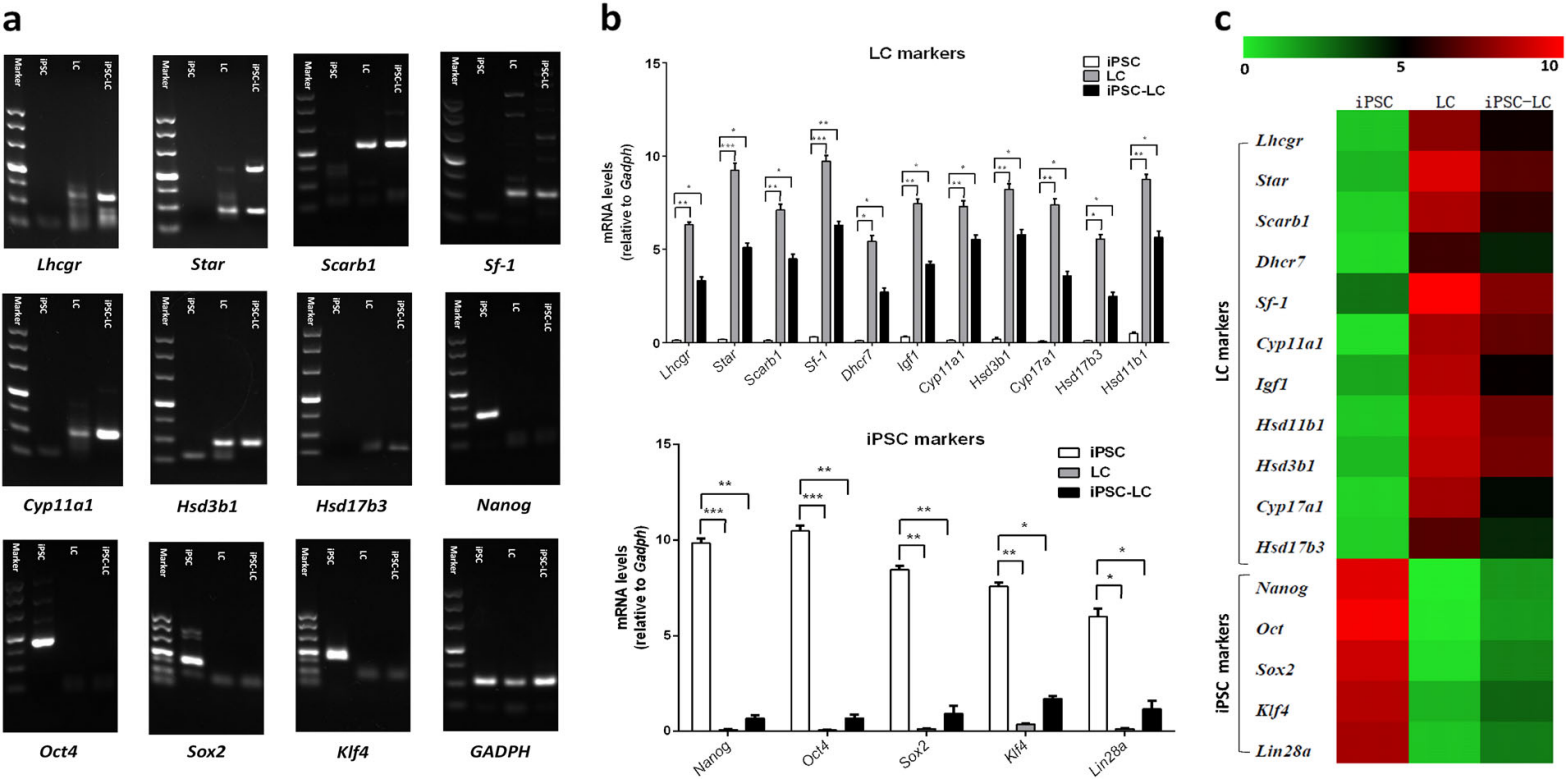
Identification of Leydig-like cells derived from induced pluripotent stem cells (iPSC-LCs) by gene expression assays. **a** The detection of expressions of Leydig cell or iPS cell relative genes using RT-PCR in iPSCs, LCs, and iPSC-LCs. **b** The comparation of expression levels of Leydig cell or iPSC relative genes using qPCR in iPSCs, LCs, and iPSC-LCs. **c** The heatmap of qPCR results (green means low expression level and red represents high expression levels). Mean ± SE, *n* = 5. ^*^P < 0.05, ^**^P < 0.01, ^***^P < 0.001 designate significant differences

After 30 day differentiation, the RT-PCR assay was also used to characterize the expressions of Leydig cell or iPS cell gene biomarkers in the enrichment iPSC-LCs. The data showed that iPSC-LCs could positively express Leydig cell gene biomarkers such as *Lhcgr*, *Star*, *Scarb1*, *Sf-1*, *Cyp11a1*, *Hsd3b1*, and *Hsd17b3*, which were required for the synthesis of steroid hormones, but not express iPS cell biomarkers such as *Nanog*, *Oct4*, *Sox2*, and *Klf4*, and this tendency is similar to LCs but is contrary to iPSCs (Fig. 3a). Meanwhile, real-time polymerase chain reaction (qPCR) assay was conducted to compare the expression levels of Leydig cell or iPS cell related genes among them. The results displayed that the expression levels of Leydig cell related genes such as *Lhcgr*, *Star*, *Scarb1*, *Sf-1*, *Dhcr7*, *Igf1*, *Cyp11a1*, *Hsd3b1*, *Cyp17a1*, *Hsd17b3*, and *HSD11b1* in iPSC-LCs were significantly lower than those of LCs but higher than those of iPSCs, which almost had no any expression. In addition, the expression levels of iPS cell related genes including *Nanog*, *Oct4*, *Sox2*, *Klf4*, and *Lin28* in iPSC-LCs and LCs were very weak, which were less than those of iPSCs (Fig. 3b). The heatmap was applied to more intuitively represent the consequences of qPCR. The green means the gene expression level is low, and the red means the gene level is high (Fig. 3c). These results also suggested that our induction method based on molecular compounds is able to differentiate iPSCs into Leydig-like cells.

### RNA-Seq and analysis

**Fig. 4:**
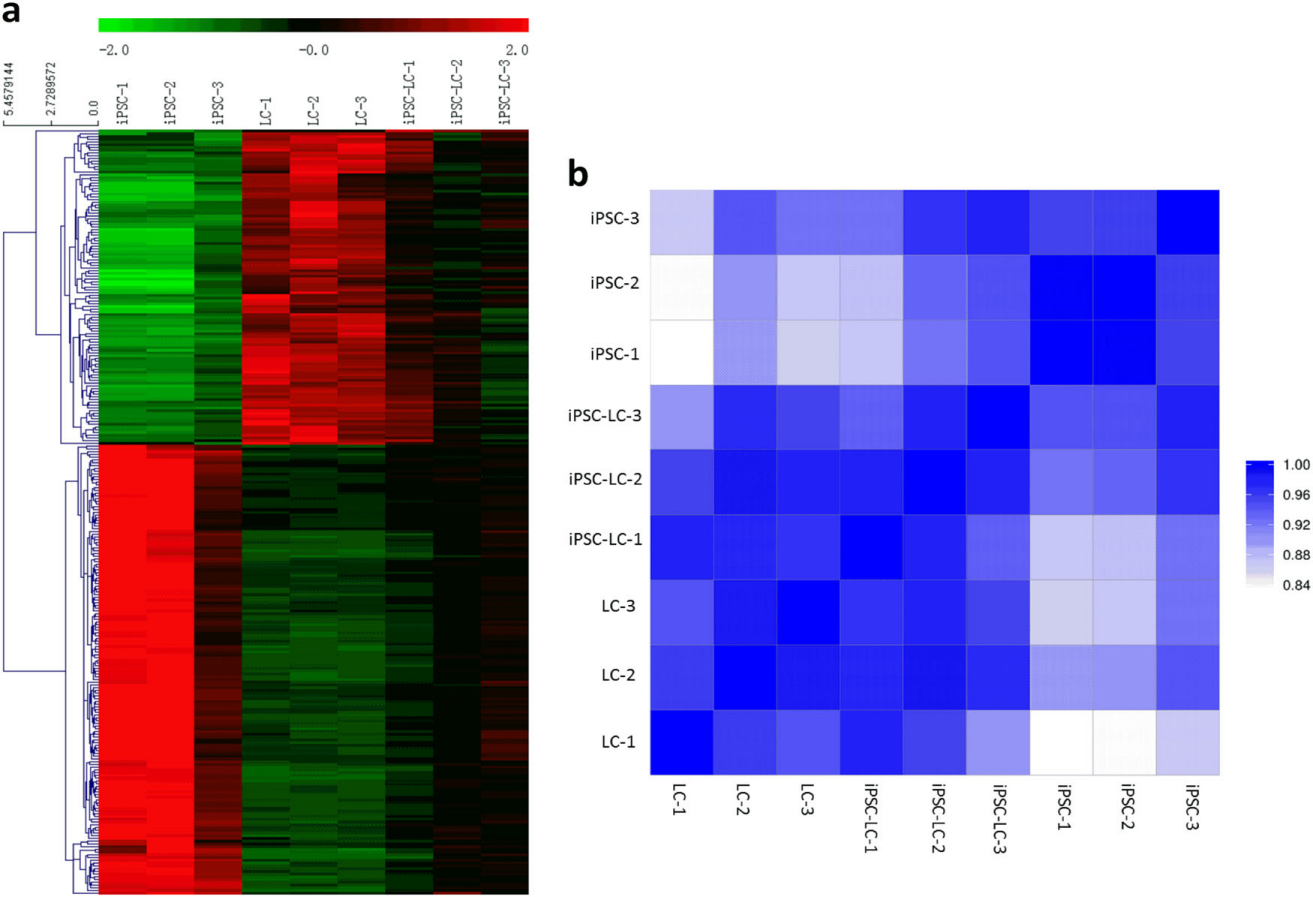
RNA-seq analysis of mRNA expressions in different groups. **a** The heatmap of mRNAs in iPSC, LC, and iPSC-LC groups (*n* = 3). Red color means up-regulated genes, Green color means downregulated genes. **b** Pearson correlation heatmap of mRNAs in iPSC, LC, and iPSC-LC groups (*n* = 3)

We further analyze the differences of genome-wide gene expression profiles in iPSCs, LCs, and iPSC-LCs through RNA-Seq assays. There were 15384 transcripts detected in these groups. Of these transcripts (iPSCs vs LCs), 5062 transcripts were significantly up-regulated (*P* < 0.05) and 4641 transcripts were significantly downregulated (*P* < 0.05). Based on these different expression transcripts, some transcripts, which could represent the special gene expressions of iPS cells and LCs were selected to produce the heatmap of mRNA expressions in these groups (Fig. 4a). These gene expression profiles in iPSCs were almost completely different from LCs, and iPSC-LCs were close to LCs although some samples were not so ideal (Fig. 4b). These results further suggested that iPSCs could be differentiated into Leydig-like cells using the induction method of molecular compounds.

### Identification of Leydig-like cells derived from iPSCs (iPSC-LCs)

**Fig. 5:**
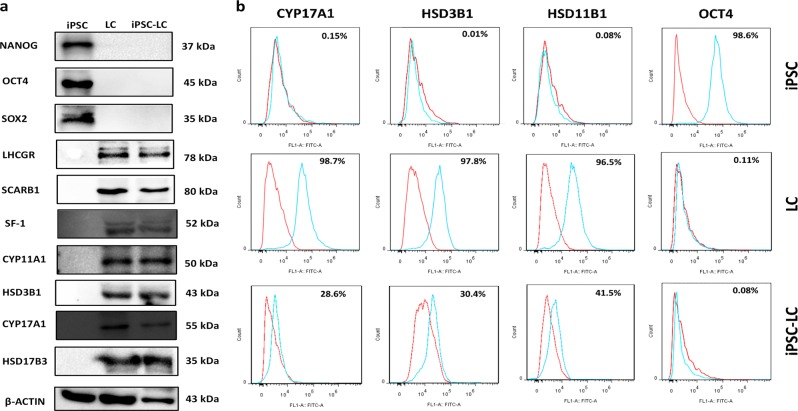
Identification of Leydig-like cells derived from induced pluripotent stem cells (iPSC-LCs) by Western blotting and flow cytometry. **a** The measurement of biomark protein expressions of Leydig cells or iPS cells using Western blotting in iPSCs, LCs, and iPSC-LCs. **b** Representative flow cytometry histograms for CYP17A1, HSD3B1, HSD11B1, and OCT4 in iPSCs, LCs, and iPSC-LCs

Western blotting assay was performed to characterize the expression of Leydig cell or iPS cell protein biomarkers in the enrichment iPSC-LCs. The data exhibited that iPSC-LCs could positively express Leydig cell biomarkers such as LHCGR, SCARB1, SF-1, CYP11A1, HSD3B1, CYP17B1, and HSD17B3, which were androgen biosynthetic enzymes for testosterone synthesis, but negatively express iPSC biomarkers NANOG, OCT4, and SOX2. These protein expressions of undifferentiated iPSCs were opposite with iPSC-LCs, and LCs were in consistent with iPSC-LCs (Fig. 5a).

The examination of representative flow cytometry histograms revealed the population expression levels of Leydig cell biomarkers CYP11A1, HSD3B1, HSD11B1, and iPS cell biomarker OCT4 in iPSC-LCs, LCs, and iPSCs after differentiation for 30 days. iPSC-LCs expressed LC-like populations with CYP17A1 (28.6%), HSD3B1 (30.4%), and HSD11B1 (41.5%), whereas they expressed iPSC populations with OCT4 (0.08%). These properties were similar to those in LCs, which expressed CYP17A1 (98.7%), HSD3B1 (97.8%), HSD11B1 (96.5%), and OCT4 (0.11%), but were different to those in iPSCs, which expressed CYP17A1 (0.15%), HSD3B1 (0.01%), HSD11B1 (0.08%), and OCT4 (98.6%) (Fig. 5b).

Taken together, these results illustrated that our differentiation protocol based on molecular compounds is able to differentiate iPSCs into Leydig-like cells.

### Transplantation of Leydig-like cells derived from iPSCs (iPSC-LCs) into the testes of rats with EDS treatment

**Fig. 6:**
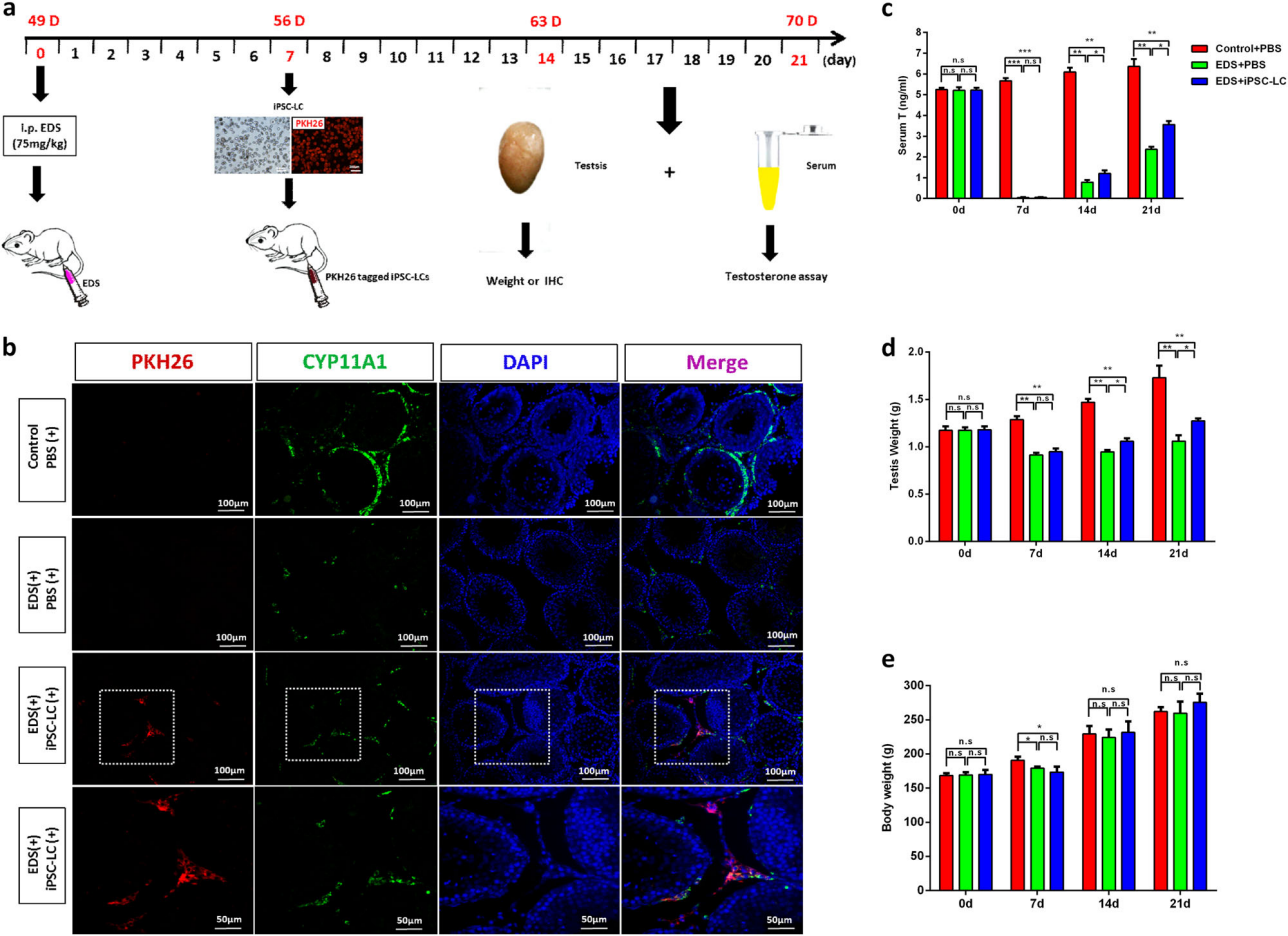
Transplantation of Leydig-like cells derived from induced pluripotent stem cells (iPSC-LCs) into the testes of rats with EDS treatment. **a** The schematic illustration of experimental procedure used for cell transplantation. **b** Immunofluorescent staining showed the accumulation of cells were positive for PKH26 (red) and CYP11A1 (green) in the interstitial area of testes at 2 weeks after grafting. Nuclei were stained with DAPI (blue). The bottom panels showed higher-magnification images of the dotted boxes in the lower-magnification images. Control/PBS( + ), rats without EDS treatment received PBS injection 7 days later; EDS( + )/PBS( + ), EDS-treated rats received PBS injection 7 days later; EDS( + )/iPSC-LCs( + ), EDS-treated mice received iPSC-LC transplantation 7 days later. **c** Serum testosterone (T) levels at different time points by radioimmunoassay. **d** The testes weights were assayed at different time points. **e** The body weights of the testes were assayed at different time points. Mean ± SE, *n* = 5. ^*^P < 0.05, ^**^P < 0.01, ^***^P < 0.001 designate significant differences. n.s > 0.05 designates no significant difference

To investigate whether iPSC-LCs have the ability to survive and function in vivo, we transplanted these cells into the parenchyma at the cranial pole of the rat testes treated with EDS 7 days later. A single injection of EDS (75 mg/kg) could specifically eliminate the LCs in adult rat testes and led to a dramatic decline in serum testosterone levels^26,27^. iPSC-LCs labeled with PKH26 (a red fluorescent dye) were implanted into recipient rats. At 0, 7, 14, and 21 days after EDS treatment, the serum and the testes were collected for analyses (Fig. 6a). Fourteen days after cell transplantation, PKH26-labeled iPSC-LCs (red) were localized exclusively to the interstitium of the testis, and expressed the LC-specific marker CYP11A1. In EDS-treated rats with phosphate-buffered saline (PBS) injection, the CYP11A1-positive cells were almost not observed in the interstitium, but rats without EDS administration strongly expressed CYP11A1 (Fig. 6b). Moreover, after EDS treatment, the concentration of serum testosterone were dramatically decreased to undetectable levels on day 7 and recovered gradually, indicating that EDS specifically eliminated the testosterone-producing LCs in the adult rat model. In the EDS-treated rat model, serum testosterone began to increase on day 14, which was recovered to ~20% of control rats. Notably, the serum testosterone levels of EDS-treated rats with iPSC-LC transplantation were higher than that of EDS-treated rats with PBS injection, but were even lower than that of rats with PBS injection on day 14 and 21 (Fig. 6c). Testosterone has an important role in maintaining the normal weights of reproductive organs^28^. At 21 days after exposure to EDS, the weights of the reproductive testes were dramatically decreased in the EDS‐treated rats, but this decrease was rescued in the iPSC-LC transplanted rats. Quantitative analysis showed that the absolute weights of the testes were significantly higher in EDS-treated rats with iPSC-LC transplantation compared with the EDS-treated rats with PBS injection on day 14 and 21, but both were lower than that of rats with PBS injection on day 7, 14, and 21 (Fig. 6d). At 21 days after exposure to EDS, the body weights of rats were significantly decreased just on day 7. Subsequently, the body weights in iPSC-LC transplanted rats or PBS injected rats started restore, and had no significantly different with PBS injected rats without EDS treatment (Fig. 6e). These results demonstrated that the transplanted iPSC-LCs in rats have acquired key properties of LCs as they have the potential to restore the serum testosterone levels of EDS-treated rat testes.

### Enumeration of CYP11A1-positive Leydig cell number

All LCs were identified by CYP11A1 because this enzyme begins expressed in all Leydig cell lineages. Brown cytosolic staining in the testis interstitium shows CYP11A1-positive cells. At 14 or 21 days after exposure to EDS, the CYP11A1-positive staining cells were observed in both groups, and the numbers of CYP11A1-positive staining cells in iPSC-LC transplantation group were more than those of PBS injection group (Fig. S2a). In addition, the quantification analysis showed that the cell numbers of iPSC-LC transplantation group more than PBS injection group on day 14 were almost similar to those of on day 21 (Fig. S2b). These data suggested that the increasing CYP11A1-positive cells might be mainly derived from the endogenous regenerated LCs, and iPSC-LCs almost had no effects on the endogenous LCs.

## Discussion

In this study, we report a novel protocol, to our knowledge, which is the first to demonstrate that iPSCs were able to be differentiated into testosterone-producing Leydig-like cells by few defined molecular compounds. In addition, when transplanted into the testes of LC-depleted rat model, iPSC-LCs could survive in the interstitium and have the potential capacity to partially recover serum testosterone production. Our findings provide new insight into stem cell-derived Leydig cell replacement therapies for the treatment of the patients with testosterone deficiency or decline.

Hypogonadism, which is also known as testosterone deficiency, affects ~ 30% of men aged 40–79 years and increases in prevalence with age^29^. Testosterone replacement therapy is a straightforward treatment for male hypogonadism, but comes with the potential risks and complications^8,30^. Therefore, researchers have sought to develop alternative treatment methods for the long‐lasting delivery of androgens. Among them, the use of stem cell-derived Leydig cell transplantation will provide a new strategy for treating the testosterone deficiency. Stem cells are gaining attentions in the regenerative medicine especially after the establishment of human ESCs in 1998^31^ and human iPSCs in 2007^32^. Human iPSCs are considered to possess similar characteristics to human ESCs^32^. As iPSCs can address the immunological and ethical problems associated with human ESCs, they represent potentially effective approach to cell transplantation-based regenerative medicine.

In our team previous review, we had systematically documented the effects of different factors such as leukemia inhibitor factor, desert hedgehog (DHH), platelet-derived growth factors, kit ligand (c-kit), insulin-like growth factor 1 (IGF1), transforming growth factor β, Activin, fibroblast growth factor 2 (FGF2), LH, androgen, and others on the development (differentiation and proliferation) of stem LCs in the LC lineages^33^. Based on this review and practical experience, we optimized several factors to induce the differentiation of iPSCs into Leydig-like cells, and the necessity and optimum dose of these defined factors were also screened and analyzed through comparing the secreted testosterone levels of iPSC-LCs in this study.

In the testis, DHH, secreted by Sertoli cells, is critical for LC development. Rat testis with a null mutation of DHH gene (*Dhh*) lacks ALCs^34^. When added to cultured seminiferous tubules during weeks 2 and 3, which LC differentiation occurs, SAG (an agonist of DHH) and Li resulted in at least 10-fold stimulation of testosterone production^35^. 22R-hydroxycholesterol (22R-OHC), a membrane-permeant cholesterol analog, can promote the enzymatic activity of CYP11A1 to synthetize steroid hormones of LCs^36^. The combined utilization of SAG, 22R-OHC, and Li at the appropriate concentrations, could mimic the effect of transcription factor SF-1 on the development of LCs. SF-1 is an orphan nuclear receptor that belongs to the NR5A subfamily, which is essential for sexual differentiation and formation of the primary steroidogenic tissues^37^. SF-1 knockout mice completely lack adrenal glands and gonads, and die soon after birth^38^. During the first phase of differentiation from day 0 to 7, SAG, 22R-OHC, and Li were added into the iPSC-DIM to induce the differentiation of iPSCs toward steroid-like cells.

In addition, PDGF receptor a is expressed in the LC lineage cells. PDGF-AA could not only significantly stimulate the proliferation of LCs^35^, but also stimulate the differentiation of LCs^39^. FGF2 belongs to a heparin-binding growth factor family. It can affect multiple cell functions, including proliferation, migration, survival, and differentiation. FGF2 can dramatically stimulate LC proliferation^35^. FGF2 can also stimulate ILC steroidogenesis in the absence of LH, and FGF2 has a biphasic effect on LH binding to its receptors (LHCGR) in ILCs, with low concentrations (0.1–1.0 ng/ml) inhibitory and high concentrations (10–100 ng/ml) stimulatory^40^. During the second phase from day 7 to 10, PDGF-AA and FGF2 were added into the iPSC-DIM to mainly promote the proliferation of the differentiated cells.

Besides cell proliferation, IGF1 also stimulates LC differentiation. Deletion of *Igf1* gene significantly delayed the maturation of LCs, with significantly decreased expression of CYP11A1, HSD3B, CYP17A1, and HSD17B3^41^. Androgen receptor (NR3C4) is a nuclear receptor and is expressed in LC lineage and other testicular cells such as Sertoli cells and peritubular Myoid cells^42^. Androgen is still important to the development of LCs^43^. During the third phase from day 10 to 17, PDGF-AA, IGF1, and Androgen were added into the iPSC-DIM to further enhance the differentiation of iPSCs toward Leydig-like cells. Meanwhile, PDGF-AA and FGF2 were once more added into the iPSC-DIM to promote the proliferation of differentiated cells during the fourth phase from day 17 to 20.

LCs are the only cells that respond to LH in the testis. LH binds to the LHCGR in LCs, resulting in both acute- and trophic effects. Acute effects involve the mobilization and delivery of cholesterol to mitochondrion to start the steroidogenesis. The trophic effects involve increases in gene transcriptions and steroidogenic enzyme activities. Both effects are required for the maintenance of an optimal steroidogenesis in the LCs^44^. Without LH stimulation, LC steroidogenic enzyme activities are reduced. LH signaling is critical to both LC differentiation and proliferation^33^. Retinoic acid (RA) is well known to influence stem cell differentiation^45^ and steroidogenesis^46,47^. RA also stimulates testosterone secretion from human fetal testis organ culture over a short period of time^48^. cAMP, as a second messenger, is known to induce steroidogenesis in a number of steroidogenic cell lines^23^. The transcriptional activity of SF-1 can be dramatically upregulated by the cAMP protein kinase A signal pathway^49^. 8-bromoadenosine 3′, 5′-cyclic monophosphate (8-Br-cAMP) is a membrane-permeable cAMP analog. Previous works had reported that SF-1 could initiate the differentiation of ESCs into steroid-producing cells with the help of 8-Br-cAMP or RA in the presence of 20α-hydroxycholesterol as a substrate^19,50^. Therefore, during the fifth phase from day 20 to 25, we added LH, RA, and 8-Br-cAMP into the iPSC-DIM to promote the maturity of differentiated cells.

In this study, we demonstrated that iPSCs could be differentiated into Leydig-like cells using the defined molecular compounds, which expressed membrane receptor: LHCGR, cholesterol transporter: SCARB1 and STAR, and steroidogenic enzymes: CYP11A1, HSD3B1, CYP17A1, and HSD17B3, had the similar gene expression profiles with LCs, and produced testosterone. Moreover, the result of flow cytometry showed that the HSD11B1 positive cells reached up to 41.5% in differentiated cells. Compared with other organs, testis is immunologically privileged^51^. To investigate whether the iPSC-LCs have the ability to survive and function in the interstitium of rat testes in vivo, we transplanted the iPSC-LCs into an EDS‐treated rat model, an androgen deficiency model, as previously described^52^. EDS is an alkylating agent, which has selective pro-apoptotic effects on LCs^27^. Approximately 2–3 weeks after a single dose of EDS, newly regenerated LCs could be observed within the testicular interstitium^53^. Approximately 8–10 weeks later, the LC population returned to its original size and had restored its ability to produce testosterone^54^. Based on these results, we collected the testes of the cell‐transplanted rats 21 days after EDS administration, at which time point the regenerated LCs appear a little, to observe the state of the transplanted cells. As a result, we observed that the transplanted PKH26-labeled iPSC-LCs localized exclusively to the interstitium of the testis, and expressed the LC-specific marker CYP11A1. Importantly, these PKH26-labeled iPSC-LCs could survive at least 2 weeks in vivo, which demonstrated that they successfully integrated into the host niche. In addition, the serum testosterone concentration of the iPSC-LC transplanted rats remained higher than that of the EDS‐treated rats up to 21 days. As testosterone plays an important role in maintaining normal reproductive organs^55^, testosterone deficiency can cause atrophy of reproductive organs^8^. At 21 days after exposure to EDS, the weights of EDS‐treated rat testes were dramatically decreased. By day 14 after transplantation, the weights of testes were significantly rescued in the iPSC-LC transplanted rats. At 21 days after exposure to EDS, the rat body weights were notably decreased just on day 7. Subsequently, the body weights in iPSC-LC transplanted rats or PBS injected rats started restore, and had no significantly different with PBS injected rats without EDS treatment on day 14 and 21.

In conclusion, we first developed a novel differentiation protocol based on molecular compounds but not bringing in the exogenous transcription factors to induce the differentiation of iPSCs into Leydig-like cells (iPSC-LCs) expressing Leydig cell lineage-specific biomarkers, having the similar gene expression profiles with LCs, and acquiring testosterone synthesis abilities. In addition, when iPSC-LCs labeled with lipophilic red dye (PKH26) were transplanted into rat testes with EDS treatment, they could survive and function in the interstitium of testes, and accelerate the recovery of serum testosterone levels and testis weights, so these findings will lay the safer groundwork for further clinical application of stem cell-derived LCs for hypogonadism.

## Materials and methods

### Culture of human iPSCs

The commercial human iPSCs used in this study were purchased from Saibei Biotechnology (Beijing, China). iPSCs (HiPSC-U1) were reprogrammed from human urine-derived cells of a 37-year-old male by the integration-free CytoTune™-iPS 2.0 Sendai Reprogramming Kit (Thermo Fisher Scientific, MA, USA). iPSCs were cultured in 1% Matrigel-coated (BD Biosciences Co., Ltd., NM, USA) Petri dishes with E8 medium (Saibei Biotechnology) at 37 °C and 5% CO_2_. The medium was refreshed daily. iPSCs were passaged once every 6 days with 0.25% ethylenediaminetetraacetic acid (EDTA, Saibei Biotechnology). 1 mL of 0.25% EDTA was added and the cells were placed at 37 °C and 5% CO_2_ for 5 min. When iPSC colonies appeared white, the solution was gently removed, and the iPSCs were washed with Mg^2+^ and Ca^2+^-free Dulbecco’s PBS (Sigma, MO, USA). iPSCs were then harvested by gently pipetting 7–10 times with 1 mL E8 medium and seeded onto fresh six-well culture plates that were coated with 1% Matrigel at a ratio of 1:6. 10 μm Y-27632 (Sigma) was supplemented in the medium on the first day of passage.

### Isolation and culture of human Leydig cells (LCs)

Human LCs were obtained from nine male donors with a mean age of 45 years old through testes excision within 20 h. Informed consent was obtained from each donor, and this study was approved by Human Research and Ethical Committee of Wenzhou Medical University. The testes were used to isolate ILCs. ILCs express all androgen biosynthetic enzymes^56^, and are capable of proliferation and differentiation^57^. The isolation of ILCs was performed as previously described^56^. In brief, the testes were perfused with collagenase (Sigma) via the testicular artery, and digested with M-199 buffer (Gibco, NY, USA) containing collagenase (0.25 mg/ml) and DNase (0.25 mg/mL, Sigma) for 15 min. Then, the cell suspension was filtered through 100 µm nylon mesh and the cells were separated by a Percoll gradient (Sigma). The cells with the density of 1.07–1.088 g/ml were collected. The purity of ILCs was evaluated by immunohistochemical staining HSD3B1, the biomarker of ILCs, as previously described^58^. The HSD3B1 staining solution contained with 0.4 mm etiocholanolone (Sigma) as the steroid substrate and NAD^+^ as a cofactor^58^. The purity of ILCs was > 95%.

The isolated ILCs were directly seeded into wells in the 24-well culture plates with the density of 2 × 10^4^ cells/well and incubated at a 37 °C, 5% CO_2_ incubator. The culture medium (LC-Medium) contains DMEM/F12 (Gibco), 5% fatal bovine serum (FBS, Gibco), 2.5% horse serum (HS, Gibco), and 1% penicillin/streptomycin (P/S, Gibco). In order to get ALCs, the culture medium were changed into differentiation-induced medium (DIM) contains DMEM/F12, 5 mm ITS (insulin, transferrin, and selenium, Sigma), 5 ng/ml luteinizing hormone (LH, PeproTech, NJ, USA), and 5 mm lithium chloride (Li, Sigma) as our team previous report^35^.

### Animal

The Sprague-Dawley rats (at 5 weeks of age) and immune deficiency (SCID) mice (at 5 weeks of age) were obtained from the laboratory animal center of Wenzhou Medical University, Wenzhou, China. All animals were kept under conditions with controlled temperature (23 ± 2 °C), a 12 h dark/light cycle, and relative humidity of 45–55%. The standard drinking water and rodent diet were accessed ad libitum. All surgical procedures and postoperative care were approved by the Wenzhou Medical University’s Animal Care and Use Committee, and were performed in accordance with the Guide for the Care and Use of Laboratory Animals.

### Differentiation of human iPSCs into Leydig-like cells (iPSC-LCs)

The point at which iPSCs were expanded to ~70% confluency in the E8 medium was defined as day −2, and at this point when iPSCs were changed into E7 medium (no FGF2) for 2 days to prepare differentiation. From day −5 to 0, the medium was refreshed daily. Prior to the beginning of differentiation, iPSCs were cultured in a differentiation-inducing medium composed of DMEM/F12, 1% bovine serum albumin (BSA) (Sigma), 5 mm ITS, 5 ng/mL LH. From 0–7 days, 0.2 μm SAG (DHH agonist, Sigma), 5 μm 22R-OHC (Steraloids, RI, USA), and 5 mm Li were added into iPSC-DIM. From 7–10 days, 5 ng/mL PDGF-AA (Sigma) and 5 ng/mL FGF2 (Sigma) were added into iPSC-DIM. From 10–17 days, 5 ng/mL PDGF-AA, 5 nM IGF1 (Sigma), and 10 μm Androgen (Sigma) were added into iPSC-DIM. From 17–20 days, 10 ng/mL PDGF-AA and 10 ng/mL FGF2 were added into iPSC-DIM. From 20–25 days, 5 ng/mL LH, 0.5 mm retinoic acid (RA, Sigma) and 1 mm 8-Br-cAMP (Sigma) were added into iPSC-DIM. From day 0 to 25, the medium was changed every 2 days by fresh iPSC-DIM. From 25–30 days, the cells were mechanically enriched by scraping away clonal iPSC-like cells. The remaining Leydig-like cells were kept in Enrichment Medium contained DMEM/F12, 5% FBS, 2.5% HS, 1 × sodiumpyruvate (Invitrogen), 1 × GlutaMAX (Invitrogen), and 1% P/S for the subsequent assays. The medium was changed every 2 days by fresh Enrichment Medium.

### Transmission electron microscopy

For TEM, the cells in different groups were prefixed with 2.5% glutaraldehyde in 0.1 m PBS for 24 h at 4 °C. Then, they were washed with PBS, and post-fixed with 1% osmium tetroxide. After gradient dehydration of acetone, they were embedded in Araldite M (Sigma Aldrich). Ultrathin sections (1 µm) were subsequently cut with an ultramicrotome, mounted on nickel grids, and stained with uranyl acetate and lead citrate. At last, the samples were sent to the electron microscope room at Wenzhou Medical University for subsequent processing and testing using a transmission electron microscope (H-600A-2; Hitachi, Tokyo, Japan).

### Testosterone measurement by radioimmunoassay

The cell culture supernatants and serum were collected at each experimental time point for the quantitative measurement of testosterone. For the cell culture supernatants, 10 ng/mL LH was in advance at least 3 h added into the medium (just having DMEM/F12) to stimulate the testosterone production of LCs or iPSC-LCs. Testosterone levels were measured with a tritium-based radioimmunoassay using anti-testosterone antibody as previously described^59^. Standards ranging between 10 and 2000 pg/mL testosterone were prepared in triplicate. Standards and samples were incubated with tracer and antibody at 4 °C overnight and charcoal-dextran suspension was used to separate the bound and free steroids. The bound steroids were mixed with a scintillation buffer and counted in a β scintillation counter (PE, CA, USA). The minimum detectable concentration for testosterone was 5 pg/mL. Quality control samples contain 100 pg/mL testosterone. The intra-assay and inter-assay coefficients of variation were within 10%.

### Immunofluorescence assays

Immunofluorescence was used to identify iPSC-LCs as a previous report^60^. In brief, after fixation with 4% paraformaldehyde (Sigma) for 15 min, cells were washed three times with PBS. Then cells were permeabilized with 0.1% TritonX-100 in PBS for 15 min at room temperature, and incubated with 3% (w/v) BSA in PBS for 1 h at room temperature. The cells were then incubated with primary antibodies as Table S1 overnight at 4 °C, and then with fluorescein isothiocyanate (FITC)-conjugated anti-mouse, FITC-conjugated anti-rabbit, Cy3-conjugated anti-mouse, and Cy3-conjugated anti-rabbit IgG secondary antibodies (1:1000, Bioword, USA) for 60 min at room temperature. Then the cells were rinsed three times with PBS thrice for 5 min each and then incubated for 15 min with DAPI (Sigma) for nuclear staining and washed three times with PBS before examination by an inverted fluorescence microscope (OLYMPUS, Japan).

### RT-PCR and qPCR

Total RNA from the cells was extracted using Trizol reagent (Invitrogen, CA, USA) according to the manufacturer’s instruction. The RNA was reversely transcribed into cDNA using the Superscript II kit (Invitrogen). The cDNAs templates were diluted 1:10, which were used to perform RT-PCR and qPCR to analyze the gene expressions. RT-PCR was performed using an authorized thermal cycler (Eppendorf, Hamburg, GER). After amplification, 1 μL of 6 × Loading buffer and 5 μL of each PCR product were mixed and electrophoresed on a 2% agarose containing 0.5 μg/mL ethidium bromide. Gels were scanned for further analysis. qPCR was performed using the Thunderbird SYBR qPCR Mix (Takara, Tokyo, Japan) according to the manufacturer’s instructions. Signals were detected using a Light Cycler 480 Detection System (Roche, Basel, Switzerland). The relative expression of genes was normalized to GAPDH. The melting curve was examined for the quality of PCR amplification for each sample, and quantification was performed using the comparative 2^-ΔΔCt^ method. The primer sequences were shown in Table S2.

### RNA-Seq analysis

Total RNA from each sample was extracted using Trizol reagent (Invitrogen, CA, USA). 1–2 μg total RNA was used to prepare the sequencing library. To sequence the libraries, the barcoded libraries were mixed, denatured to single stranded DNA, captured on Illumina flow cell, amplified in situ, and subsequently sequenced for 150 cycles for both ends on Illumina HiSeq 4000 instrument. Sequence quality was examined using the FastQC software. The transcript abundances for each sample were estimated with StringTie, and the FPKM value for gene and transcript level were calculated with R package Ballgown. The differentially expressed genes and transcripts were filtered using R package Ballgown. The correlation analysis was based on gene expression levels. Hierarchical Clustering, Gene Ontology, Pathway analysis, scatter plots and volcano plots were performed with the differentially expressed genes in R, Python for statistical computing and graphics^61^.

### Western blotting

Cells were washed with cold PBS and were lysed in 1 × radioimmunoprecipitation assay lysis buffer in the presence of a protease inhibitor mixture/1% phosphatase inhibitor mixture (Roche). 50 μg of protein samples were applied to a 10% sodium dodecyl sulfate polyacrylamide gel electrophoresis and transferred into the polyvinylidene difluoride membranes (Sigma) by an electroblot apparatus. After being blocked with a blocking solution (5% fat-free milk) for 2 h at 4 °C, the membranes were incubated with primary antibodies as Table S1 in the blocking solution at 4 °C overnight. The membranes were washed with tris-buffered saline with Tween 20 (TBST) five times (10 min each), and incubated with horseradish peroxidase-conjugated secondary antibody (1:3000, Bioword) at room temperature for 2 h. The membranes were then washed five times (10 min each) with TBST. Bands were visualized with enhanced chemiluminescence (ECL, Pierce, USA). The protein expression was normalized to β-actin.

### Flow cytometry

The cell samples were fixed with 4% paraformaldehyde in PBS and permeabilized with 0.1% TritonX-100 (Sigma). The samples were then labeled with primary or isotype control antibodies for 30 min at 4 °C. Primary and isotype control antibodies that were not conjugated to fluorophores were labeled with fluorophore-conjugated secondary antibody for 30 min at 4 °C. The labeled samples were detected by flow cytometry analyzer (BD, USA). Data analysis was performed on FCS Express 4 Flow Research Edition software.

### Tagging iPSC-derived Leydig-like cells (iPSC-LCs) with PKH26

The standard protocol was conducted as PKH26 Product Information Sheet (Sigma, MINI2). In brief, the suspension containing 2 × 10^7^ cells were centrifuged (400 × *g*, 5 min) and were washed once using fresh medium without serum. After centrifuging, the supernatant was removed and no more than 25 μL of 2 × Cell Suspension was prepared by adding 1 mL of Diluent C, the cells were resuspended with gentle pipetting to ensure complete dispersion. 2 × Dye Solution (4 × 10^–6^ m) was prepared by adding 4 μL of the PKH26 ethanolic dye solution to 1 mL of Diluent C and mixed well. Then 1 mL of 2 × Dye Solution was rapidly added into the 1 mL of 2 × Cell Suspension. Final concentration after mixing was 2 × 10^–6^ m PKH26 with 1 × 10^7^ cells/well. The mixing suspension was incubated at room temperature for 5 min with periodic mixing. The action of the staining was stopped by adding an equal volume (2 mL) of serum. Then the suspension was centrifuged at 400 × *g* for 10 min and washed three times. Finally, the cells tagged with PKH26 were seeded on fresh wells and used for injection.

### Transplantation of iPSC-derived Leydig-like cells (iPSC-LCs) in vivo

For evaluating whether iPSC-LCs could facilitate the recovery of LC dysfunction of rats, iPSC-LC transplantation was performed as previously described with some modifications^62^. Sixty 49-days-old male Sprague-Dawley rats (*n* = 5 for each group at each time point) were used in this study. Before transplantation, male rats were administered a single intraperitoneal injection of EDS (75 mg/kg, Pterosaur Biotech Co., Ltd., Hangzhou, China), which was dissolved in dimethyl sulfoxide (Sigma): H_2_O (1: 3, v/v). This treatment resulted in the elimination of LCs in the adult testes of rats^63^. Then iPSC-LCs labeled with PKH26 (red) were resuspended manually, and harvested in a 15 mL Falcon tube. Cells were rinsed twice with PBS following centrifugation at 200 × *g* for 5 min. Finally each pellet was resuspended in PBS for transplantation. Cells were loaded into a 1 mL syringe for injection into the testis of adult Sprague-Dawley male rats that had been treated with EDS. Approximately 2 × 10^6^ PKH26-labeled iPSC-LCs in 40 mL of PBS were injected into the parenchyma of recipient testes 7 days after the rats received EDS. The control animals for the experimental group were EDS-treated rats that had received a testicular injection of the PBS vehicle. Testes from all animals were examined at 0, 7, 14, and 21 days after EDS treatment.

### Immunohistochemistry

One testis from each rat was used for immunohistochemistry (Vector Laboratories, Inc., Burlingame, CA, USA) according to the manufacturer’s instructions. The rats were killed with an overdose of sodium pentobarbital (Sigma). Testes were removed and fixed in 4% paraformaldehyde overnight at 4 °C. Then testes were dehydrated with a graded series of ethanol and xylene and subsequently embedded in paraffin. Five micrometer-thick transverse sections (5 μm) were cut, de-waxed in water, and were mounted on glass slides. Antigen retrieval was performed by microwave irradiation for 10 min in 10 mm (pH 6.0) of citrate buffer, after which endogenous peroxidase was blocked with 0.5% of H_2_O_2_ in methanol for 30 min. Some sections were fixed with 4% paraformaldehyde for 15 min and washed 3 times with PBS. Then they were permeabilized with 0.1% TritonX-100 in PBS for 15 min at room temperature, and incubated with 3% (w/v) BSA (Sigma) in PBS for 1 h at room temperature. Then these sections were then incubated with an CYP11A1 polyclonal antibody diluted 1:1000 for 2 h at room temperature, and then with FITC-conjugated IgG secondary antibodies (1:1000, Bioword) for 1 h at room temperature. These sections were rinsed with PBS three times for 5 min each time. Then the sections were incubated for 15 min with DAPI (10 μg/mL, Sigma) for nuclear staining and washed three times with PBS. The sections were cover-slipped with resin (Thermo Fisher Scientific, Waltham, UK). At last, they were examined by an inverted fluorescence microscope (OLYMPUS). The cells with CYP11A1 staining in the interstitial area represent the LC^64^.

Other sections were directly incubated with CYP11A1 polyclonal antibody diluted 1:1000, for 2 h at room temperature. Diaminobenzidine was used for visualizing the antibody–antigen complexes, positive labeling LCs by brown cytoplasmic staining. Mayer hematoxylin was applied in counterstaining. The sections were then dehydrated in graded concentrations of alcohol and cover-slipped with resin (Thermo Fisher Scientific, Waltham, UK). Lastly, they were examined by a fluorescence microscope (LEICA). The cells with CYP11A1 staining in the interstitial area represent the LCs.

### Teratoma analysis

For teratoma formation, iPSCs (5 × 10^6^ cells) were dissociated with 0.5 mm EDTA, centrifuged, resuspended in 100 μL E8 with 1% Matrigel, and injected into the hind limbs of 6-week-old male SCID mice. Teratomas were collected after 6 weeks, and fixed in 4% paraformaldehyde for paraffin embedding and hematoxylin and eosin staining. Slides were imaged and analyzed by a qualified clinical pathologist.

### Karyotype analysis

The division of iPSCs was blocked with 50 µg/mL of colcemid solution (Invitrogen, USA). Cells were washed with PBS and harvested with trypsin at room temperature for 2 min. Then cells were fixed in methanol/glacial acetic acid (3:1) for three times and dropped onto slides for chromosome spreads. At last, the slides were baked at 55 °C for overnight. Standard G-banded karyotypes were obtained using Giemsa solution staining (Giemsa, Japan).

### Enumeration of Leydig cell number by stereology

To enumerate CYP11A1-positive Leydig cell numbers, sampling of the testis was performed according to a fractionator method as our previous report^65^. Identification of all Leydig cell lineages was done by the staining of CYP11A1. About 10 testis sections per rat were sampled from each testis. The total number of LCs was calculated by multiplying the number of LCs counted in a known fraction of the testis by the inverse of the sampling probability.

### Statistical analysis

All experiments were performed at least thrice, and the data are presented as the mean ± standard error of the mean. Statistical analyses were evaluated using an unpaired Student’s *t* test or one-way analysis of variance for more than two groups. *P* < 0.05 was considered statistically significant.

## Supplementary information

Figure S1

Figure S2

Supplementary Information

Supplementary figure legends
