# Supplementary material for: Differentiation of human induced pluripotent stem cells into Leydig-like cells with molecular compounds
Source: Cell Death Dis. 2019 Mar 4;10(3):220. doi: 10.1038/s41419-019-1461-0 (PMC6399252; doi:10.1038/s41419-019-1461-0)
Supplement: Supplementary file 3 — Supplementary Information [file 41419_2019_1461_MOESM3_ESM.doc]

**Supplementary table S1. Antibodies**

| **Antibody** | **Species** | **Vendor (City, State, catalogue)** | **Dilution** | | |  | |  |
| --- | --- | --- | --- | --- | --- | --- | --- | --- |
|  |  |  | **WB** | **IC FC** | | **IF** | | |
| β-ACTIN | rabbit | Cell Signaling Technology (Danvers,  MA,12620) | 1:1000 | ND | ND | | ND | |
| LHCGR | mouse | Abcam (San Francisco, CA, ab204950) | 1:1000 | ND | ND | | ND | |
| SCARB1  SF-1 | rabbit  mouse | Abcam (San Francisco, CA,  ab217318)  Santa Cruz (Santa Cruz, CA,  sc-393592) | 1:1000  1:200 | ND  ND | ND  ND | | ND  ND | |
| CYP11A1 | rabbit | Abcam (San Francisco, CA,  ab75497) | 1:1000 | 1:500 | ND | | 1:500 | |
| HSD3B1 | mouse | Abcam (San Francisco, CA,ab55268) | 1:1000 | ND | 1:500 | | 1:200 | |
| CYP17A1 | rabbit | Abcam (San Francisco, CA, ab125022) | 1:2000 | ND | 1:500 | | ND | |
| HSD11B1 | goat | Abcam (San Francisco, CA,  Ab27501) | 1:2000 | ND | 1:500 | | ND | |
| HSD17B3 | rabbit | Abcam (San Francisco, CA,ab126228) | 1:2000 | ND | ND | | 1:500 | |
| NANOG | rabbit | Abcam (San Francisco, CA, ab106465) | 1:1000 | ND | ND | | 1:200 | |
| OCT4 | rabbit | Abcam (San Francisco, CA, ab181557) | 1:1000 | ND | 1:200 | | 1:250 | |
| SOX2  SSEA4 | rabbit  Mouse | Cell Signaling technology(23064) Abcam (San Francisco, CA, ab16287) | 1:1000  1:1000 | ND  ND | ND  ND | | ND  ND | |

ND = Not detected; WB = Western blot; IC =  Immunohistochemistry; FC = Flow Cyt; IF = Immunofluorescence.

**Supplementary table S2**. Primer information

| **Primer**  **Symbol** | **Gene name** | **Primer direction** | **Sequences (5’to 3’)** | **PCR**  **(bp)** | **Accession** |
| --- | --- | --- | --- | --- | --- |
| Lhcgr | Luteinizing hormone receptor | Forward | CACATAACCACCATACCAGGAAA | 124 | [NM_000233.3](https://www.ncbi.nlm.nih.gov/entrez/viewer.fcgi?db=nucleotide&id=189409126) |
|  |  | Reverse | AAGTCAGTGTCGTCCCATTGA |  |  |
| Scarb1 | Scavenger receptor class B, member 1 | Forward | GTCGCAGGCATTGGACAAAC | 220 | [NM_001082959.1](https://www.ncbi.nlm.nih.gov/entrez/viewer.fcgi?db=nucleotide&id=132566679) |
|  |  | Reverse | CAGGACCTTGGCTCCGGATT |  |  |
| Star | Steroidogenic acute regulatory protein | Forward | GGGAGTGGAACCCCAATGTC | 78 | [NM_000349.2](https://www.ncbi.nlm.nih.gov/entrez/viewer.fcgi?db=nucleotide&id=56243550) |
|  |  | Reverse | CCAGCTCGTGAGTAATGAATGT |  |  |
| Sf-1  Cyp11a1 | Nuclear receptor  steroidogenic factor 1  Cholesterol side chain cleavage enzyme | Forward  Reverse  Forward | GGAGGCTTGCGAAGGAGAAG  AGCTTACCCAACGGCGTG  GCAGTGTCTCGGGACTTCG | 105  102 | [NM_001178030.1](https://www.ncbi.nlm.nih.gov/entrez/viewer.fcgi?db=nucleotide&id=295842329)  [NM_001099773.1](https://www.ncbi.nlm.nih.gov/nuccore/NM_001099773.1) |
|  |  | Reverse | GGCAAAGCGGAACAGGTCA |  |  |
| Hsd3b1 | 3β-Hydroxysteroid dehydrogenase 1 | Forward | CACATGGCCCGCTCCATAC | 90 | [NM_000862.2](https://www.ncbi.nlm.nih.gov/entrez/viewer.fcgi?db=nucleotide&id=115345345) |
|  |  | Reverse | GTGCCGCCGTTTTTCAGATTC |  |  |
| Cyp17a1 | P450 17α-hydroxylase/ 17,20-lyase | Forward | TATGGCCCCATCTATTCGGTT | 161 | NM_000102.3 |
|  |  | Reverse | GCGATACCCTTACGGTTGTTG |  |  |
| Hsd17b3 | 17β-Hydroxysteroid dehydrogenase 3 | Forward | GTCAACAATGTCGGAATGCTTC | 91 | [NM_000197.1](https://www.ncbi.nlm.nih.gov/entrez/viewer.fcgi?db=nucleotide&id=4557648) |
|  |  | Reverse | TGATGTTACAATGGATGAGGCTC |  |  |
| Dhcr7 | 7-dehydrocholesterol reductase | Forward | AGGTGTGCGCAGGACTTTAG | 174 | [NM_001360.2](https://www.ncbi.nlm.nih.gov/nuccore/NM_001360.2) |
|  |  | Reverse | TGGGAATGTTGGGTTGCGAT |  |  |
| Igf1 | Insulin like growth factor 1 | Forward | AGAGCCTGCGCAATGGAATA | 166 | NM_012515.2 |
|  |  | Reverse | TTGGGTTGGAAGACTGCTGA |  |  |
| Nanog | Nanog homeobox | Forward | CAAGAACTCTCCAACATCCTGAA | 127 | NM_024865.2 |
|  |  | Reverse | CCTGCGTCACACCATTGCTATTC |  |  |
| Oct4 | Organic cation/carnitine transporter4 | Forward | GAAGGATGTGGTCCGAGTGT | 183 | NM_001173531.2 |
|  |  | Reverse | GTGAAGTGAGGGCTCCCATA |  |  |
| Sox2 | SRY (sex determining region Y)-box 2 | Forward | CAGGAGTTGTCAAGGCAGAGA | 171 | NM_003106.3 |
|  |  | Reverse | CCGCCGCCGATGATTGTTA |  |  |
| Klf4 | Kruppel like factor 4 | Forward | GCCGCTCCATTACCAAGAG | 166 | NM_004235.4  NM_024674.5  [NM_001256799.2](https://www.ncbi.nlm.nih.gov/nuccore/NM_001256799.2) |
| Lin28a  GADPH | Lin-28 homolog A  Glyceraldehyde-3-phosphate dehydrogenase | Reverse  Forward  Reverse  Forward  Reverse | GTGTGCCTTGAGATGGGAAC  GGGGCCTTCCATGTAGAAAGT  GGAGTTACATTCGTTCGTCTCCT  ACAACTTTGGTATCGTGGAAGG  GCCATCACGCCACAGTTTC | 152  101 |  |

**Supplementary table S3. The necessity of factors was analyzed through comparing secreted testosterone levels of iPSC-LCs**.

| **SAG** | **22R-OH** | **Li** | **PDGF-AA** | **FGF2** | **IGF-1** | **Androgen** | **LH** | **RA** | **88r-cAMP** | **T level of iPSC-LCs** |
| --- | --- | --- | --- | --- | --- | --- | --- | --- | --- | --- |
| + | + | + | + | + | + | + | + | + | + | A |
| - | + | + | + | + | + | + | + | + | + | F |
| + | - | + | + | + | + | + | + | + | + | F |
| + | + | - | + | + | + | + | + | + | + | F |
| + | + | + | - | + | + | + | + | + | + | E |
| + | + | + | + | - | + | + | + | + | + | B |
| + | + | + | + | + | - | + | + | + | + | C |
| + | + | + | + | + | + | - | + | + | + | C |
| + | + | + | + | + | + | + | - | + | + | F |
| + | + | + | + | + | + | + | + | - | + | D |
| + | + | + | + | + | + | + | + | + | - | D |

**Note: T level A (0.5-0.6 ng/mL); B (0.4-0.5 ng/mL); C (0.3-0.4 ng/mL); D (0.2-0.3 ng/mL); E (0.1-0.2 ng/mL); F (0-0.1 ng/mL); “+”means adding; “-” means minusing.**

**Supplementary table S4. The optimum dose of factors was screened by through comparing secreted testosterone levels of iPSC-LCs**.

| **Reagent** | **The optimum concentration (range tested)** | **T level of iPSC-LCs** |
| --- | --- | --- |
| SAG | 0.2 μM (0.1–1) | A |
| 22R-OHC | 5 μM (1–10) | A |
| Lithium (Li) | 5 mM (1–10) | A |
| PDGF-AA | 10 ng/mL (5–20) | A |
| FGF2 | 10 ng/mL (5–20) | A |
| IGF-1 | 5 nM (1–10) | A |
| Androgen | 10 μM (5–20) | A |
| LH | 5 ng/mL (1–10) | A |
| Retinoic acid (RA) | 0.5 mM (0.1-1) | A |
| 8Br-cAMP | 1 mM (0.1–2) | A |

**Note: T level A (0.5-0.6 ng/mL); B (0.4-0.5 ng/mL); C (0.3-0.4 ng/mL); D (0.2-0.3 ng/mL); E (0.1-0.2 ng/mL); F (0 -0.1 ng/mL).**
