# Supplementary material for: Differentiation of human induced pluripotent stem cells into Leydig-like cells with molecular compounds
Source: Cell Death Dis. 2019 Mar 4;10(3):220. doi: 10.1038/s41419-019-1461-0 (PMC6399252; doi:10.1038/s41419-019-1461-0)
Supplement: Supplementary file 4 — Supplementary figure legends [file 41419_2019_1461_MOESM4_ESM.doc]

**Fig. S1 Identification of induced pluripotent stem cells (iPSCs). a** Clonal morphology of iPSCs under inverted microscope. **b** The Karyotype analysis of iPSCs. **c** Three germ layers (endoderm, ectoderm, and mesoderm) from teratoma of iPSCs.

**Fig. S2 Immunohistochemical staining of CYP11A1 in rat testis sections in different groups on day 14 and 21.** **a** CYP11A1 staining in rat testis sections in different groups on day 14 and 21. **b** The quantification of CYP11A1 positive cell numbers in rat testis sections in different groups on day 14 and 21. Mean ± SE, n=10. *^**^P*<0.01,*^***^P*<0.001 designate significant differences.
